# Supplementary material for: An unexpected noncarpellate epigynous flower from the Jurassic of China
Source: eLife. 2018 Dec 18;7:e38827. doi: 10.7554/eLife.38827 (PMC6298773; doi:10.7554/eLife.38827)
Supplement: Supplementary file 1. [file elife-38827-supp1.docx]

| \| Specimen \| Drawer \| Top view \| Bottom view \| Side view \| Associated plants \| \| --- \| --- \| --- \| --- \| --- \| --- \| \| BP22213 \| D2 \|  \|  \|  \| *?Hymenophyllaceae* \| \| BP22214 \| D2 \|  \|  \|  \| *Clathropteris* \| \| BP22215 \| D2 \|  \|  \|  \| *Podozamites* \| \| BP22216 \| D2 \|  \|  \|  \| *Baiera asadai* \| \| BP22217 \| D2 \|  \|  \|  \| *Cladophlebis* \| \| BP22218 \| D2 \|  \|  \|  \| *Ginkgoites sibiricus* \| \| BP22219 \| D2 \|  \|  \|  \| *Cladophlebis* \| \| BP22220 \| D3 \|  \| 8 \|  \|  \| \| BP22221A \| D4 \| 3 \| 24 \|  \|  \| \| BP22221B \| D4 \| 1 \| 4 \|  \|  \| \| BP22222A \| D5 \| 40 \| 9 \| 2 \|  \| \| BP22222B \| D5 \| 7 \| 27 \| 2 \|  \| \| BP22222C \| D5 \|  \| 8 \|  \|  \| \| BP22222D \| D5 \| 7 \| 2 \|  \|  \| \| BP22223 \| D3 \| 3 \| 13 \| 1 \| *Ptilophyllum* \| \| BP22224 \| D5 \|  \| 1 \| 4 \|  \| \| BP22225A \| D4 \| 1 \| 1 \|  \| *Clathropteris, Desmiophyllum?* \| \| BP22225B \| D4 \|  \| 1 \| 1 \| *Nilssoniopteris vittata, Nilssonia parabrevis, Clathropteris* \| \| BP22226 \| D5 \| 6 \| 1 \|  \| *Nilssonia parabrevis* \| \| BP22227 \| D4 \|  \|  \| 1 \|  \| \| BP22227A \| D7 \| 1 \| 4 \|  \|  \| \| BP22227B \| D7 \| 1 \|  \|  \| *Ptilophyllum* \| \| BP22228 \| D3 \| 50 \| 1 \|  \|  \| \| BP22230 \| D3 \|  \|  \|  \| *Nilssoniopteris vittata, Nilssonia parabrevis* \| \| BP22231 \| D3 \|  \|  \|  \| *Desmiophyllum?* \| \| BP22232 \| D3 \|  \|  \|  \| *Nilssoniopteris vittata, Clathropteris* \| \| BP22233 \| D3 \|  \|  \|  \| *Clathropteris meniscioides,* Ferns \| \| BP22234A、B \| D2 \|  \|  \|  \| *Neocalamites horridus* \| \| BP22235 \| D3 \|  \|  \|  \| *Neocalamites horridus* \| \| BP22236 \| D1 \|  \|  \|  \| *Ptilophyllum contiguum* \| \| BP22237 \| D1 \|  \|  \|  \| *Nilssoniopteris vittata, Clathropteris meniscioides, Desmiophyllum?* \| \| BP22238 \| D1 \|  \|  \|  \| *Nilssoniopteris vittata, Ptilophyllum hsingshanense, Conites* \| \| BP22239 \| D1 \|  \|  \|  \| *Spiropteris* \| \| BP22240 \| D1 \|  \| 1 \|  \| *Nilssoniopteris vittata, Ptilophyllum hsingshanense,* Ferns \| \| BP22241 \| D4 \|  \|  \|  \| *Nilssonia parabrevis, Conites* \| \| BP22242A \| D4 \|  \| 1 \|  \| *Nilssonia parabrevis, Conites* \| \| BP22242B \| D4 \|  \|  \|  \| *Nilssonia parabrevis, Conites* \| \| BP22243A \| D7 \| 1 \| 1 \|  \|  \| \| BP22243B \| D7 \| 1 \| 1 \|  \| *Coniopteris szeiana, Desmiophyllum?* \| \| BP22244A \| D7 \|  \|  \|  \| *Coniopteris,* Ferns \| \| BP22244B \| D7 \|  \|  \|  \| *Ferns* \| \| BP22245 \| D7 \|  \|  \|  \| *Nilssonia parabrevis, Clathropteris* \| \| BP22246 \| D7 \| 1 \|  \|  \|  \| \| BP22247A \| D7 \|  \| 1 \|  \|  \| \| BP22247B \| D7 \| 1 \|  \|  \|  \| \| BP22248A \| D7 \|  \|  \|  \| *Swedenborgia* \| \| BP22248B \| D7 \|  \|  \|  \| *Otozamites* \| \| BP22249 \| D7 \|  \|  \|  \| *Swedenborgia* \| \| BP22250 \| D6 \|  \|  \|  \| *Pterophyllum* \| \| BP22251 \| D6 \|  \|  \|  \| *Desmiophyllum?, Cladophlebis,Baiera* \| \| BP22252 \| D6 \|  \|  \|  \| *Danaeopsis, Baiera* \| \| BP22253 \| D6 \|  \|  \|  \| *Nilssoniopteris?* \| \| BP22254 \| D6 \|  \|  \|  \| *Ferns* \| \| BP22255 \| D6 \|  \|  \|  \| *Danaeopsis* \| \| BP22256 \| D6 \|  \| 1 \|  \| *Desmiophyllum?* \| \| BP22257 \| D6 \|  \| 2 \|  \|  \| \| BP22258 \| D6 \|  \| 1 \|  \|  \| \| BP22259 \| D6 \|  \| 1 \|  \|  \| \| BP22260 \| D6 \| 1 \|  \|  \|  \| \| BP22261 \| D6 \|  \|  \|  \| *Neocalamites horridus* \| \| BP22262 \| D6 \|  \|  \|  \| *Nilssonia parabrevis, Desmiophyllum?* \| \| BP22489 \| D3 \|  \| 1 \| 1 \| *Nilssonia parabrevis, Desmiophyllum?* \| \| PB22278 \| D8 \| 1 \| 2 \|  \| *Nilssonia parabrevis, Desmiophyllum?, fern* \| \| PB22279 \| D8 \|  \| 1 \|  \|  \| \| PB22280 \| D8 \| 2 \| 3 \|  \| *Nilssonia parabrevis, Desmiophyllum?* \| \| PB22281 \| D8 \|  \|  \| 1 \| *Nilssoniopteris vittata, Desmiophyllum?* \| \| PB22282 \| D8 \|  \| 1 \| 1 \| *Nilssonia parabrevis, Desmiophyllum?* \| \| **TOTAL** \|  \| 128 \| 122 \| 14 \| 264 \| |
| --- | --- | --- | --- | --- | --- | --- | --- | --- | --- | --- | --- | --- | --- | --- | --- | --- | --- | --- | --- | --- | --- | --- | --- | --- | --- | --- | --- | --- | --- | --- | --- | --- | --- | --- | --- | --- | --- | --- | --- | --- | --- | --- | --- | --- | --- | --- | --- | --- | --- | --- | --- | --- | --- | --- | --- | --- | --- | --- | --- | --- | --- | --- | --- | --- | --- | --- | --- | --- | --- | --- | --- | --- | --- | --- | --- | --- | --- | --- | --- | --- | --- | --- | --- | --- | --- | --- | --- | --- | --- | --- | --- | --- | --- | --- | --- | --- | --- | --- | --- | --- | --- | --- | --- | --- | --- | --- | --- | --- | --- | --- | --- | --- | --- | --- | --- | --- | --- | --- | --- | --- | --- | --- | --- | --- | --- | --- | --- | --- | --- | --- | --- | --- | --- | --- | --- | --- | --- | --- | --- | --- | --- | --- | --- | --- | --- | --- | --- | --- | --- | --- | --- | --- | --- | --- | --- | --- | --- | --- | --- | --- | --- | --- | --- | --- | --- | --- | --- | --- | --- | --- | --- | --- | --- | --- | --- | --- | --- | --- | --- | --- | --- | --- | --- | --- | --- | --- | --- | --- | --- | --- | --- | --- | --- | --- | --- | --- | --- | --- | --- | --- | --- | --- | --- | --- | --- | --- | --- | --- | --- | --- | --- | --- | --- | --- | --- | --- | --- | --- | --- | --- | --- | --- | --- | --- | --- | --- | --- | --- | --- | --- | --- | --- | --- | --- | --- | --- | --- | --- | --- | --- | --- | --- | --- | --- | --- | --- | --- | --- | --- | --- | --- | --- | --- | --- | --- | --- | --- | --- | --- | --- | --- | --- | --- | --- | --- | --- | --- | --- | --- | --- | --- | --- | --- | --- | --- | --- | --- | --- | --- | --- | --- | --- | --- | --- | --- | --- | --- | --- | --- | --- | --- | --- | --- | --- | --- | --- | --- | --- | --- | --- | --- | --- | --- | --- | --- | --- | --- | --- | --- | --- | --- | --- | --- | --- | --- | --- | --- | --- | --- | --- | --- | --- | --- | --- | --- | --- | --- | --- | --- | --- | --- | --- | --- | --- | --- | --- | --- | --- | --- | --- | --- | --- | --- | --- | --- | --- | --- | --- | --- | --- | --- | --- | --- | --- | --- | --- | --- | --- | --- | --- | --- | --- | --- | --- | --- | --- | --- | --- | --- | --- | --- | --- | --- | --- | --- | --- | --- | --- | --- | --- | --- | --- | --- | --- | --- | --- | --- | --- | --- | --- | --- | --- | --- | --- | --- | --- | --- | --- | --- | --- | --- | --- | --- | --- | --- | --- | --- | --- | --- | --- | --- | --- | --- | --- |
