# Supplementary material for: An unexpected noncarpellate epigynous flower from the Jurassic of China
Source: eLife. 2018 Dec 18;7:e38827. doi: 10.7554/eLife.38827 (PMC6298773; doi:10.7554/eLife.38827)
Supplement: Supplementary file 2. — Online Information 3D virtual image of the holotype of Nanjinganthus. Click on the link, using ctrl/shift and mouse, you can manipulate the image for your observation. [file elife-38827-supp2.docx]

| Spot | Pb | U | Th/U | Isotopic rations | | | | | | Ages(Ma) | | | | | |  |
| --- | --- | --- | --- | --- | --- | --- | --- | --- | --- | --- | --- | --- | --- | --- | --- | --- |
|  | (ppm) | |  | ^207^Pb/^206^Pb | err% | ^207^Pb/^235^U | err% | ^206^Pb/^238^U | err% | ^207^Pb/^235^U | 1σ | ^206^Pb/^238^U | 1σ | ^206^Pb/^238^U | 1σ | Concordance |
| 16NJ09.1 | 42 | 519 | 0.866 | 0.0982 | 1.54 | 1.0345 | 1.63 | 0.0764 | 1.04 | 721 | 12 | 475 | 5 | 1591 | 29 | 48.02% |
| 16NJ09.2 | 2 | 22 | 0.86 | 0.0961 | 7.80 | 1.3940 | 7.68 | 0.1052 | 1.39 | 886 | 68 | 645 | 9 | 1550 | 146 | 62.51% |
| 16NJ09.4 | 3 | 37 | 0.905 | 0.1255 | 7.13 | 1.1696 | 7.21 | 0.0676 | 1.75 | 786 | 57 | 422 | 7 | 2035 | 126 | 13.54% |
| 16NJ09.5 | 58 | 233 | 0.827 | 0.1241 | 1.51 | 3.9634 | 1.61 | 0.2316 | 0.99 | 1627 | 26 | 1343 | 13 | 2016 | 27 | 78.85% |
| 16NJ09.6 | 5 | 88 | 2.32 | 0.0529 | 6.68 | 0.3087 | 6.82 | 0.0423 | 1.15 | 273 | 19 | 267 | 3 | 324 | 152 | 97.79% |
| 16NJ09.7 | 6 | 122 | 1.099 | 0.0930 | 3.61 | 0.5546 | 3.64 | 0.0433 | 1.14 | 448 | 16 | 273 | 3 | 1487 | 68 | 35.95% |
| 16NJ09.8 | 137 | 312 | 0.493 | 0.1543 | 1.54 | 8.2808 | 1.65 | 0.3891 | 0.99 | 2262 | 37 | 2119 | 21 | 2395 | 26 | 93.23% |
| 16NJ09.10 | 12 | 234 | 1.14 | 0.0769 | 3.17 | 0.4537 | 3.29 | 0.0428 | 1.04 | 380 | 12 | 270 | 3 | 1119 | 63 | 59.34% |
| 16NJ09.11 | 16 | 198 | 0.662 | 0.0786 | 2.20 | 0.7851 | 2.21 | 0.0724 | 1.00 | 588 | 13 | 451 | 4 | 1163 | 44 | 69.46% |
| 16NJ09.12 | 166 | 305 | 0.348 | 0.2010 | 1.51 | 13.7308 | 1.65 | 0.4955 | 1.02 | 2731 | 45 | 2594 | 27 | 2834 | 25 | 94.72% |
| 16NJ09.13 | 57 | 874 | 0.647 | 0.1002 | 1.55 | 0.8013 | 1.63 | 0.0580 | 0.99 | 598 | 10 | 363 | 4 | 1628 | 29 | 35.55% |
| 16NJ09.14 | 107 | 1038 | 0.892 | 0.1103 | 1.56 | 1.4457 | 1.62 | 0.0951 | 1.05 | 908 | 15 | 585 | 6 | 1804 | 28 | 44.90% |
| 16NJ09.16 | 62 | 748 | 0.672 | 0.0575 | 1.61 | 0.6097 | 1.71 | 0.0770 | 0.99 | 483 | 8 | 478 | 5 | 509 | 35 | 98.88% |
| 16NJ09.19 | 7 | 155 | 0.651 | 0.0531 | 3.81 | 0.3120 | 3.84 | 0.0426 | 1.03 | 276 | 11 | 269 | 3 | 334 | 86 | 97.45% |
| 16NJ09.20 | 58 | 804 | 0.458 | 0.0749 | 1.61 | 0.7146 | 1.67 | 0.0692 | 0.99 | 547 | 9 | 431 | 4 | 1066 | 32 | 73.03% |
| 16NJ09.21 | 20 | 136 | 1.223 | 0.0658 | 2.01 | 1.1598 | 2.12 | 0.1279 | 1.06 | 782 | 17 | 776 | 8 | 799 | 42 | 99.24% |
| 16NJ09.22 | 73 | 232 | 1.297 | 0.1110 | 1.53 | 4.2758 | 1.63 | 0.2794 | 0.99 | 1689 | 27 | 1588 | 16 | 1816 | 28 | 93.67% |
| 16NJ09.24 | 24 | 194 | 0.828 | 0.0825 | 1.76 | 1.2947 | 1.93 | 0.1139 | 1.12 | 843 | 16 | 695 | 8 | 1257 | 34 | 78.67% |
| 16NJ09.25 | 28 | 345 | 0.528 | 0.0583 | 1.85 | 0.6360 | 1.96 | 0.0791 | 1.01 | 500 | 10 | 491 | 5 | 542 | 40 | 98.14% |
| 16NJ09.26 | 50 | 631 | 0.998 | 0.0561 | 1.66 | 0.5357 | 1.77 | 0.0693 | 0.99 | 436 | 8 | 432 | 4 | 455 | 37 | 99.15% |
| 16NJ09.27 | 59 | 249 | 1.02 | 0.1352 | 1.51 | 3.7920 | 1.62 | 0.2034 | 0.99 | 1591 | 26 | 1194 | 12 | 2167 | 26 | 66.69% |
| 16NJ09.28 | 36 | 163 | 0.761 | 0.0773 | 1.61 | 2.0782 | 1.72 | 0.1950 | 1.01 | 1142 | 20 | 1148 | 12 | 1129 | 32 | 99.43% |
| 16NJ09.29 | 50 | 799 | 0.455 | 0.1016 | 1.55 | 0.8086 | 1.65 | 0.0577 | 1.02 | 602 | 10 | 362 | 4 | 1654 | 29 | 33.61% |
| 16NJ09.30 | 15 | 290 | 0.988 | 0.0738 | 2.67 | 0.4387 | 2.93 | 0.0431 | 1.04 | 369 | 11 | 272 | 3 | 1036 | 54 | 64.28% |
| 16NJ09.32 | 101 | 719 | 0.297 | 0.0789 | 1.56 | 1.5277 | 1.64 | 0.1404 | 1.10 | 942 | 15 | 847 | 9 | 1169 | 31 | 88.85% |
| 16NJ17.1 | 0 | 2 | 8.658 | 0.0712 | 2.16 | 1.7990 | 2.44 | 0.1832 | 1.03 | 1045 | 25 | 1084 | 11 | 964 | 21 | 96.37% |
| 16NJ17.2 | 0 | 2 | 2.388 | 0.0803 | 2.97 | 1.0193 | 3.42 | 0.0917 | 1.17 | 714 | 24 | 565 | 7 | 1205 | 36 | 73.78% |
| 16NJ17.3 | 0 | 1 | 2.03 | 0.0730 | 3.49 | 1.3998 | 3.62 | 0.1392 | 0.86 | 889 | 32 | 840 | 7 | 1014 | 35 | 94.17% |
| 16NJ17.4 | 1 | 5 | 1.006 | 0.1122 | 2.52 | 1.5091 | 3.17 | 0.0967 | 1.20 | 934 | 30 | 595 | 7 | 1835 | 46 | 42.96% |
| 16NJ17.5 | 3 | 12 | 0.221 | 0.1323 | 2.18 | 5.0590 | 2.67 | 0.2773 | 1.42 | 1829 | 49 | 1578 | 22 | 2129 | 46 | 84.06% |
| 16NJ17.6 | 1 | 6 | 0.072 | 0.1057 | 2.62 | 1.7656 | 2.99 | 0.1207 | 0.73 | 1033 | 31 | 735 | 5 | 1726 | 45 | 59.43% |
| 16NJ17.7 | 452 | 233 | 0.4 | 0.1629 | 2.30 | 9.6964 | 2.43 | 0.4321 | 0.54 | 2406 | 59 | 2315 | 12 | 2486 | 57 | 96.07% |
| 16NJ17.8 | 196 | 102 | 0.848 | 0.1628 | 3.04 | 3.7322 | 3.95 | 0.1636 | 1.44 | 1578 | 62 | 977 | 14 | 2485 | 76 | 38.40% |
| 16NJ17.9 | 6 | 13 | 3.71 | 0.1662 | 2.39 | 9.8653 | 2.57 | 0.4309 | 0.72 | 2422 | 62 | 2310 | 17 | 2520 | 60 | 95.13% |
| 16NJ17.10 | 1 | 6 | 1.237 | 0.0828 | 2.61 | 1.6445 | 3.03 | 0.1434 | 0.90 | 987 | 30 | 864 | 8 | 1265 | 33 | 85.73% |
| 16NJ17.11 | 0 | 3 | 4.197 | 0.0673 | 2.60 | 0.4644 | 3.18 | 0.0497 | 1.03 | 387 | 12 | 313 | 3 | 847 | 22 | 76.10% |
| 16NJ17.12 | 0 | 3 | 1.215 | 0.0907 | 3.26 | 1.8033 | 4.23 | 0.1417 | 1.46 | 1047 | 44 | 854 | 12 | 1439 | 47 | 77.49% |
| 16NJ17.14 | 0 | 2 | 1.571 | 0.0672 | 3.50 | 0.4204 | 3.68 | 0.0453 | 0.59 | 356 | 13 | 286 | 2 | 844 | 30 | 75.24% |
| 16NJ17.15 | 4 | 9 | 3.65 | 0.1881 | 2.73 | 12.1051 | 3.12 | 0.4661 | 1.29 | 2613 | 82 | 2466 | 32 | 2725 | 75 | 94.07% |
| 16NJ17.16 | 0 | 3 | 1.471 | 0.0897 | 3.20 | 1.7950 | 3.44 | 0.1447 | 0.73 | 1044 | 36 | 871 | 6 | 1419 | 45 | 80.21% |
| 16NJ17.17 | 1 | 5 | 2.129 | 0.1019 | 2.97 | 3.2330 | 3.12 | 0.2300 | 0.54 | 1465 | 46 | 1334 | 7 | 1658 | 49 | 90.19% |
| 16NJ17.18 | 5 | 15 | 4.975 | 0.1168 | 2.75 | 5.1458 | 2.98 | 0.3191 | 0.87 | 1844 | 55 | 1786 | 16 | 1908 | 52 | 96.74% |
| 16NJ17.19 | 2 | 5 | 5.775 | 0.1118 | 2.60 | 4.9003 | 2.76 | 0.3179 | 0.64 | 1802 | 50 | 1780 | 11 | 1828 | 48 | 98.72% |
| 16NJ17.20 | 2 | 7 | 10.43 | 0.1221 | 2.48 | 4.9544 | 2.68 | 0.2942 | 0.72 | 1812 | 49 | 1662 | 12 | 1987 | 49 | 91.03% |
| 16NJ17.21 | 3 | 9 | 2.782 | 0.1117 | 2.40 | 5.3689 | 2.58 | 0.3487 | 0.65 | 1880 | 48 | 1928 | 13 | 1827 | 44 | 97.49% |
| 16NJ17.22 | 0 | 3 | 1.563 | 0.0767 | 3.00 | 1.3475 | 3.47 | 0.1267 | 0.89 | 866 | 30 | 769 | 7 | 1112 | 33 | 87.35% |
| 16NJ17.23 | 1 | 7 | 2.336 | 0.0764 | 2.40 | 1.6207 | 2.59 | 0.1538 | 0.58 | 978 | 25 | 922 | 5 | 1106 | 27 | 93.94% |
| 16NJ17.24 | 1 | 5 | 6.116 | 0.0768 | 2.39 | 1.2516 | 2.90 | 0.1182 | 1.39 | 824 | 24 | 720 | 10 | 1115 | 27 | 85.57% |
| 16NJ17.25 | 7 | 24 | 2.457 | 0.1407 | 2.36 | 5.7386 | 2.55 | 0.2961 | 0.68 | 1937 | 49 | 1672 | 11 | 2236 | 53 | 84.12% |
| 16NJ17.26 | 3 | 10 | 3.035 | 0.1130 | 2.28 | 4.9338 | 2.55 | 0.3169 | 0.96 | 1808 | 46 | 1774 | 17 | 1849 | 42 | 98.10% |
| 16NJ17.27 | 2 | 9 | 2.316 | 0.0956 | 2.24 | 2.9837 | 2.52 | 0.2261 | 0.79 | 1403 | 35 | 1314 | 10 | 1540 | 35 | 93.19% |
| 16NJ17.28 | 0 | 1 | 2.878 | 0.1004 | 3.05 | 1.9117 | 5.82 | 0.1338 | 3.85 | 1085 | 63 | 809 | 31 | 1631 | 50 | 65.94% |
| 16NJ17.29 | 0 | 2 | 1.794 | 0.0835 | 2.79 | 1.9537 | 3.76 | 0.1680 | 1.65 | 1100 | 41 | 1001 | 17 | 1280 | 36 | 90.14% |
| 16NJ17.31 | 7 | 20 | 1.833 | 0.1378 | 2.11 | 6.3608 | 2.33 | 0.3346 | 0.77 | 2027 | 47 | 1861 | 14 | 2200 | 46 | 91.08% |
| 16NJ17.32 | 4 | 8 | 4.552 | 0.1685 | 2.10 | 11.0648 | 2.24 | 0.4763 | 0.55 | 2529 | 57 | 2511 | 14 | 2543 | 53 | 99.31% |
| 16NJ17.34 | 2 | 7 | 2.996 | 0.0884 | 2.16 | 2.8030 | 2.30 | 0.2299 | 0.52 | 1356 | 31 | 1334 | 7 | 1392 | 30 | 98.34% |
| 16NJ17.35 | 4 | 10 | 1.65 | 0.1123 | 2.15 | 5.7354 | 2.40 | 0.3705 | 0.91 | 1937 | 46 | 2032 | 18 | 1837 | 39 | 95.33% |
| 16NJ17.36 | 2 | 7 | 3.382 | 0.1370 | 2.19 | 6.4169 | 2.32 | 0.3399 | 0.53 | 2035 | 47 | 1886 | 10 | 2190 | 48 | 92.12% |
| 16NJ17.37 | 0 | 2 | 1.564 | 0.0908 | 2.83 | 2.2141 | 3.43 | 0.1759 | 1.26 | 1186 | 41 | 1044 | 13 | 1443 | 41 | 86.48% |
| 16NJ17.38 | 0 | 4 | 2.009 | 0.0766 | 2.70 | 0.8340 | 3.09 | 0.0787 | 0.76 | 616 | 19 | 488 | 4 | 1110 | 30 | 73.89% |
| 16NJ17.39 | 7 | 13 | 1.709 | 0.1702 | 2.48 | 11.0912 | 2.65 | 0.4728 | 0.57 | 2531 | 67 | 2496 | 14 | 2560 | 63 | 98.60% |
| 16NJ17.40 | 2 | 10 | 9.706 | 0.1131 | 2.63 | 3.7761 | 2.87 | 0.2423 | 0.82 | 1588 | 46 | 1399 | 12 | 1849 | 49 | 86.48% |
| 16NJ17.41 | 2 | 6 | 5.233 | 0.1108 | 2.60 | 5.1871 | 2.75 | 0.3398 | 0.56 | 1851 | 51 | 1886 | 11 | 1813 | 47 | 98.13% |
| 16NJ17.42 | 0 | 2 | 45.9 | 0.0724 | 2.55 | 0.8759 | 2.72 | 0.0879 | 0.83 | 639 | 17 | 543 | 5 | 998 | 25 | 82.40% |
| 16NJ17.43 | 0 | 4 | 3.102 | 0.0820 | 2.90 | 0.5287 | 3.45 | 0.0464 | 1.04 | 431 | 15 | 293 | 3 | 1245 | 36 | 52.67% |
| 16NJ17.44 | 0 | 2 | 1.336 | 0.1360 | 2.92 | 1.9353 | 4.03 | 0.1017 | 1.93 | 1093 | 44 | 624 | 12 | 2177 | 63 | 24.80% |
| 16NJ17.45 | 11 | 23 | 1.064 | 0.1512 | 2.20 | 8.8030 | 2.35 | 0.4224 | 0.56 | 2318 | 54 | 2272 | 13 | 2360 | 52 | 97.96% |
| 16NJ17.46 | 1 | 3 | 4.937 | 0.1303 | 2.16 | 6.7052 | 2.37 | 0.3734 | 0.81 | 2073 | 49 | 2045 | 17 | 2102 | 45 | 98.64% |
| 16NJ17.48 | 0 | 2 | 0.299 | 0.0774 | 3.28 | 0.7370 | 3.40 | 0.0696 | 1.49 | 561 | 19 | 434 | 6 | 1131 | 37 | 70.69% |
| 16NJ17.49 | 2 | 8 | 0.231 | 0.0945 | 2.13 | 2.9850 | 2.29 | 0.2291 | 0.59 | 1404 | 32 | 1330 | 8 | 1517 | 32 | 94.44% |
| 16NJ17.50 | 1 | 4 | 0.737 | 0.0761 | 2.76 | 1.4112 | 3.45 | 0.1332 | 1.13 | 894 | 31 | 806 | 9 | 1099 | 30 | 89.13% |
| 16NJ17.51 | 1 | 10 | 0.848 | 0.0698 | 2.16 | 1.2866 | 2.35 | 0.1336 | 0.65 | 840 | 20 | 809 | 5 | 922 | 20 | 96.14% |
| 16NJ17.52 | 3 | 9 | 0.578 | 0.1185 | 2.07 | 5.8582 | 2.21 | 0.3586 | 0.56 | 1955 | 43 | 1976 | 11 | 1933 | 40 | 98.96% |
| 16NJ17.53 | 6 | 14 | 0.174 | 0.1607 | 2.05 | 9.0089 | 2.19 | 0.4065 | 0.55 | 2339 | 51 | 2199 | 12 | 2463 | 51 | 93.64% |
| 16NJ17.54 | 0 | 4 | 0.409 | 0.1019 | 2.35 | 0.9858 | 4.49 | 0.0687 | 2.87 | 697 | 31 | 428 | 12 | 1660 | 39 | 37.44% |
| 16NJ17.55 | 0 | 3 | 0.959 | 0.1180 | 3.19 | 1.4087 | 3.48 | 0.0878 | 2.34 | 893 | 31 | 542 | 13 | 1926 | 61 | 35.46% |
| 16NJ17.56 | 3 | 17 | 1.261 | 0.1123 | 2.14 | 2.1656 | 2.31 | 0.1398 | 0.63 | 1170 | 27 | 843 | 5 | 1837 | 39 | 61.25% |
| 16NJ17.57 | 2 | 4 | 0.316 | 0.1599 | 2.10 | 9.3955 | 2.27 | 0.4258 | 0.66 | 2377 | 54 | 2287 | 15 | 2455 | 52 | 96.04% |
| 16NJ17.58 | 2 | 8 | 1.575 | 0.1210 | 2.86 | 2.2623 | 3.51 | 0.1341 | 0.95 | 1201 | 42 | 811 | 8 | 1971 | 56 | 52.02% |
| 16NJ17.59 | 2 | 12 | 1.175 | 0.0717 | 2.22 | 1.2799 | 2.38 | 0.1293 | 0.54 | 837 | 20 | 784 | 4 | 978 | 22 | 93.25% |
| 16NJ17.60 | 1 | 5 | 0.896 | 0.0896 | 2.47 | 1.6763 | 2.93 | 0.1350 | 0.81 | 1000 | 29 | 816 | 7 | 1416 | 35 | 77.57% |
| 16NJ17.61 | 4 | 14 | 1.977 | 0.0967 | 2.46 | 1.8485 | 2.64 | 0.1387 | 0.87 | 1063 | 28 | 837 | 7 | 1562 | 38 | 73.03% |
| 16NJ17.62 | 1 | 4 | 0.402 | 0.0959 | 2.39 | 3.0935 | 2.57 | 0.2336 | 0.59 | 1431 | 37 | 1354 | 8 | 1546 | 37 | 94.27% |
| 16NJ17.63 | 0 | 4 | 0.176 | 0.0942 | 2.92 | 0.9776 | 3.11 | 0.0751 | 0.57 | 692 | 22 | 467 | 3 | 1512 | 44 | 51.71% |
| 16NJ17.64 | 1 | 6 | 1.001 | 0.0843 | 2.75 | 0.9164 | 3.41 | 0.0780 | 0.92 | 660 | 22 | 484 | 4 | 1299 | 36 | 63.59% |
| 16NJ17.65 | 5 | 10 | 0.46 | 0.2165 | 2.21 | 13.0890 | 2.57 | 0.4368 | 0.89 | 2686 | 69 | 2336 | 21 | 2955 | 65 | 85.03% |
| 16NJ17.66 | 0 | 5 | 0.273 | 0.1216 | 2.20 | 1.7304 | 2.52 | 0.1031 | 1.05 | 1020 | 26 | 633 | 7 | 1980 | 44 | 38.81% |
| 16NJ17.68 | 0 | 1 | 0.621 | 0.0854 | 4.30 | 0.6830 | 6.04 | 0.0560 | 1.89 | 529 | 32 | 351 | 7 | 1325 | 57 | 49.61% |
| 16NJ17.69 | 3 | 9 | 0.198 | 0.1157 | 2.06 | 5.1219 | 2.24 | 0.3211 | 0.76 | 1840 | 41 | 1795 | 14 | 1892 | 39 | 97.52% |
| 16NJ17.70 | 551 | 307 | 0.469 | 0.1287 | 2.06 | 6.6390 | 2.19 | 0.3741 | 0.52 | 2065 | 45 | 2049 | 11 | 2081 | 43 | 99.22% |
| 16NJ17.71 | 734 | 386 | 1.81 | 0.0777 | 2.12 | 1.4160 | 2.25 | 0.1322 | 0.50 | 896 | 20 | 801 | 4 | 1139 | 24 | 88.12% |
| 16NJ17.72 | 69 | 115 | 0.465 | 0.1185 | 3.16 | 1.5471 | 6.49 | 0.0899 | 3.71 | 949 | 62 | 555 | 21 | 1933 | 61 | 28.94% |
| 16NJ17.73 | 517 | 192 | 0.734 | 0.1600 | 2.09 | 8.8007 | 2.23 | 0.3991 | 0.51 | 2318 | 52 | 2165 | 11 | 2456 | 51 | 92.95% |
| 16NJ17.74 | 488 | 111 | 1.183 | 0.1991 | 2.21 | 10.8231 | 2.36 | 0.3950 | 0.70 | 2508 | 59 | 2146 | 15 | 2819 | 62 | 83.14% |
| 16NJ17.75 | 135 | 47 | 0.514 | 0.1699 | 2.54 | 8.5726 | 3.40 | 0.3617 | 1.21 | 2294 | 78 | 1990 | 24 | 2556 | 65 | 84.74% |
| 16NJ17.76 | 925 | 242 | 1.13 | 0.1605 | 2.10 | 9.2399 | 2.26 | 0.4183 | 0.51 | 2362 | 53 | 2253 | 11 | 2461 | 52 | 95.14% |
| 16NJ17.77 | 438 | 270 | 0.501 | 0.1218 | 2.15 | 5.5418 | 2.33 | 0.3307 | 0.54 | 1907 | 44 | 1842 | 10 | 1983 | 43 | 96.45% |
| 16NJ17.78 | 213 | 297 | 0.096 | 0.1223 | 2.18 | 5.2938 | 2.37 | 0.3149 | 0.67 | 1868 | 44 | 1765 | 12 | 1991 | 43 | 94.17% |
| 16NJ17.79 | 434 | 395 | 0.254 | 0.1399 | 2.23 | 5.7558 | 2.47 | 0.2994 | 0.68 | 1940 | 48 | 1688 | 11 | 2226 | 50 | 85.10% |
| 16NJ17.80 | 62 | 92 | 0.258 | 0.1148 | 2.87 | 2.3435 | 5.28 | 0.1435 | 3.10 | 1226 | 65 | 864 | 27 | 1877 | 54 | 58.18% |
| 16NJ17.81 | 125 | 267 | 0.261 | 0.0708 | 2.28 | 1.6249 | 2.45 | 0.1670 | 0.57 | 980 | 24 | 996 | 6 | 951 | 22 | 98.42% |
| 16NJ17.82 | 357 | 238 | 0.309 | 0.1632 | 2.16 | 10.1674 | 2.29 | 0.4524 | 0.56 | 2450 | 56 | 2406 | 14 | 2489 | 54 | 98.17% |
| 16NJ18.1 | 6 | 12 | 1.264 | 0.1483 | 2.38 | 8.7178 | 2.49 | 0.4253 | 0.72 | 2309 | 57 | 2285 | 17 | 2327 | 55 | 98.94% |
| 16NJ18.2 | 6 | 8 | 1.684 | 0.2359 | 2.14 | 15.0514 | 2.47 | 0.4611 | 0.84 | 2818 | 70 | 2444 | 20 | 3093 | 66 | 84.69% |
| 16NJ18.3 | 95 | 43 | 0.843 | 0.1409 | 1.96 | 7.3142 | 1.98 | 0.3764 | 0.56 | 2151 | 43 | 2060 | 12 | 2239 | 44 | 95.58% |
| 16NJ18.4 | 122 | 260 | 0.352 | 0.0771 | 1.77 | 1.6941 | 1.82 | 0.1592 | 0.56 | 1006 | 18 | 953 | 5 | 1125 | 20 | 94.36% |
| 16NJ18.5 | 182 | 495 | 0.72 | 0.0819 | 2.16 | 0.9290 | 2.16 | 0.0823 | 0.54 | 667 | 14 | 510 | 3 | 1244 | 27 | 69.11% |
| 16NJ18.6 | 430 | 421 | 0.309 | 0.1227 | 1.65 | 6.1997 | 1.74 | 0.3661 | 0.58 | 2004 | 35 | 2011 | 12 | 1996 | 33 | 99.68% |
| 16NJ18.7 | 270 | 219 | 0.511 | 0.1784 | 1.72 | 5.6482 | 1.81 | 0.2302 | 0.96 | 1923 | 35 | 1335 | 13 | 2638 | 45 | 55.96% |
| 16NJ18.9 | 32 | 50 | 1.027 | 0.1626 | 1.65 | 11.8633 | 1.76 | 0.5293 | 0.76 | 2594 | 46 | 2738 | 21 | 2483 | 41 | 94.72% |
| 16NJ18.10 | 4 | 12 | 0.49 | 0.2186 | 1.66 | 8.3691 | 2.36 | 0.2778 | 1.77 | 2272 | 54 | 1580 | 28 | 2970 | 49 | 56.24% |
| 16NJ18.11 | 1 | 5 | 0.243 | 0.1053 | 1.69 | 2.6474 | 1.77 | 0.1823 | 0.68 | 1314 | 23 | 1080 | 7 | 1720 | 29 | 78.30% |
| 16NJ18.12 | 5 | 18 | 0.757 | 0.1431 | 1.82 | 4.8941 | 1.92 | 0.2479 | 0.54 | 1801 | 35 | 1427 | 8 | 2265 | 41 | 73.81% |
| 16NJ18.13 | 0 | 1 | 0.597 | 0.0897 | 3.20 | 2.3957 | 3.35 | 0.1930 | 0.88 | 1241 | 42 | 1138 | 10 | 1420 | 45 | 90.88% |
| 16NJ18.14 | 1 | 5 | 0.461 | 0.0792 | 1.92 | 1.6867 | 1.99 | 0.1543 | 0.49 | 1004 | 20 | 925 | 5 | 1178 | 23 | 91.52% |
| 16NJ18.15 | 4 | 11 | 0.305 | 0.1565 | 2.18 | 7.3800 | 2.54 | 0.3403 | 0.67 | 2159 | 55 | 1888 | 13 | 2418 | 53 | 85.68% |
| 16NJ18.16 | 7 | 19 | 0.326 | 0.1568 | 1.81 | 7.5374 | 2.07 | 0.3478 | 0.66 | 2177 | 45 | 1924 | 13 | 2421 | 44 | 86.84% |
| 16NJ18.17 | 16 | 34 | 1.776 | 0.2739 | 1.77 | 10.5334 | 1.90 | 0.2788 | 0.64 | 2483 | 47 | 1585 | 10 | 3328 | 59 | 43.39% |
| 16NJ18.18 | 7 | 14 | 3.36 | 0.1247 | 2.01 | 2.8524 | 2.86 | 0.1642 | 1.35 | 1369 | 39 | 980 | 13 | 2025 | 41 | 60.26% |
| 16NJ18.19 | 6 | 14 | 0.712 | 0.1568 | 1.73 | 8.4745 | 2.19 | 0.3903 | 0.96 | 2283 | 50 | 2124 | 20 | 2422 | 42 | 92.52% |
| 16NJ18.20 | 1 | 6 | 0.573 | 0.1214 | 2.60 | 1.4984 | 2.92 | 0.0889 | 0.67 | 930 | 27 | 549 | 4 | 1978 | 51 | 30.70% |
| 16NJ18.21 | 4 | 12 | 0.346 | 0.1239 | 1.70 | 5.7849 | 1.87 | 0.3388 | 0.90 | 1944 | 36 | 1881 | 17 | 2014 | 34 | 96.62% |
| 16NJ18.22 | 6 | 13 | 0.37 | 0.1604 | 1.74 | 9.4081 | 1.80 | 0.4256 | 0.56 | 2379 | 43 | 2286 | 13 | 2459 | 43 | 95.94% |
| 16NJ18.23 | 0 | 3 | 0.063 | 0.0890 | 1.87 | 2.1665 | 2.03 | 0.1763 | 0.63 | 1170 | 24 | 1047 | 7 | 1404 | 26 | 88.20% |
| 16NJ18.24 | 90 | 158 | 0.185 | 0.3274 | 2.48 | 21.5134 | 3.20 | 0.4693 | 1.06 | 3162 | 101 | 2480 | 26 | 3605 | 89 | 72.51% |
| 16NJ18.27 | 12 | 18 | 2.626 | 0.3071 | 2.41 | 9.3380 | 4.55 | 0.2138 | 2.39 | 2372 | 108 | 1249 | 30 | 3506 | 84 | 10.11% |
| 16NJ18.28 | 1 | 8 | 1.191 | 0.0757 | 2.21 | 1.3632 | 2.20 | 0.1307 | 0.53 | 873 | 19 | 792 | 4 | 1087 | 24 | 89.76% |
| 16NJ18.30 | 2 | 6 | 0.33 | 0.1610 | 1.69 | 9.4860 | 1.85 | 0.4278 | 0.90 | 2386 | 44 | 2296 | 21 | 2466 | 42 | 96.07% |
| 16NJ18.31 | 8 | 15 | 1.087 | 0.1883 | 1.70 | 10.6356 | 2.07 | 0.4088 | 1.03 | 2492 | 52 | 2209 | 23 | 2728 | 46 | 87.22% |
| 16NJ18.32 | 568 | 448 | 0.363 | 0.1532 | 1.66 | 8.6438 | 1.85 | 0.4093 | 0.87 | 2301 | 43 | 2212 | 19 | 2383 | 40 | 95.95% |
| 16NJ18.33 | 118 | 57 | 0.469 | 0.1796 | 2.21 | 9.1142 | 2.56 | 0.3666 | 1.04 | 2350 | 60 | 2014 | 21 | 2649 | 59 | 83.32% |
| 16NJ18.34 | 223 | 75 | 1.416 | 0.1891 | 1.71 | 10.6518 | 1.82 | 0.4084 | 0.57 | 2493 | 45 | 2208 | 13 | 2735 | 47 | 87.06% |
| 16NJ18.35 | 32 | 40 | 0.726 | 0.1061 | 3.73 | 2.1355 | 3.79 | 0.1459 | 0.65 | 1160 | 44 | 878 | 6 | 1733 | 65 | 67.82% |
| 16NJ18.36 | 57 | 50 | 1.291 | 0.1320 | 3.23 | 2.5332 | 3.37 | 0.1388 | 0.60 | 1282 | 43 | 838 | 5 | 2125 | 69 | 47.05% |
| 16NJ18.37 | 208 | 21 | 0.878 | 0.6272 | 1.77 | 40.7307 | 2.80 | 0.4665 | 1.64 | 3789 | 106 | 2468 | 41 | 4572 | 81 | 46.50% |
| 16NJ18.39 | 163 | 619 | 0.385 | 0.1012 | 1.74 | 0.9685 | 1.81 | 0.0694 | 0.46 | 688 | 12 | 433 | 2 | 1646 | 29 | 41.01% |
| 16NJ19.1 | 65 | 72 | 0.874 | 0.1151 | 2.17 | 5.9161 | 2.18 | 0.3737 | 0.76 | 1964 | 43 | 2047 | 16 | 1882 | 41 | 95.94% |
| 16NJ19.2 | 11 | 57 | 0.438 | 0.0645 | 7.07 | 0.9697 | 7.44 | 0.1090 | 1.93 | 688 | 51 | 667 | 13 | 759 | 54 | 96.83% |
| 16NJ19.3 | 78 | 208 | 0.882 | 0.0721 | 3.29 | 1.3771 | 4.19 | 0.1372 | 1.04 | 879 | 37 | 829 | 9 | 987 | 33 | 93.92% |
| 16NJ19.4 | 168 | 242 | 2.262 | 0.0834 | 1.54 | 1.8194 | 1.67 | 0.1587 | 1.09 | 1052 | 18 | 950 | 10 | 1278 | 20 | 89.18% |
| 16NJ19.6 | 26 | 259 | 0.416 | 0.0629 | 2.65 | 0.4243 | 2.75 | 0.0489 | 0.66 | 359 | 10 | 308 | 2 | 705 | 19 | 83.29% |
| 16NJ19.7 | 18 | 207 | 0.678 | 0.0528 | 5.53 | 0.2374 | 5.60 | 0.0327 | 0.71 | 216 | 12 | 207 | 1 | 321 | 18 | 95.71% |
| 16NJ19.8 | 17 | 223 | 0.513 | 0.0562 | 4.87 | 0.2544 | 5.25 | 0.0328 | 0.75 | 230 | 12 | 208 | 2 | 460 | 22 | 89.23% |
| 16NJ19.9 | 0 | 3 | 0.958 | 0.0632 | 2.87 | 0.4375 | 3.27 | 0.0499 | 0.78 | 369 | 12 | 314 | 2 | 714 | 21 | 82.71% |
| 16NJ19.10 | 41 | 98 | 0.913 | 0.0660 | 3.86 | 1.1693 | 4.21 | 0.1278 | 0.59 | 786 | 33 | 775 | 5 | 807 | 31 | 98.55% |
| 16NJ19.11 | 57 | 69 | 1.296 | 0.1707 | 1.60 | 4.0567 | 1.76 | 0.1726 | 0.88 | 1646 | 29 | 1026 | 9 | 2564 | 41 | 39.64% |
| 16NJ19.12 | 29 | 238 | 0.253 | 0.0589 | 2.47 | 0.5702 | 2.51 | 0.0703 | 0.53 | 458 | 11 | 438 | 2 | 562 | 14 | 95.43% |
| 16NJ19.13 | 87 | 226 | 0.876 | 0.0733 | 1.61 | 1.4827 | 1.74 | 0.1467 | 0.67 | 923 | 16 | 882 | 6 | 1023 | 16 | 95.35% |
| 16NJ19.14 | 212 | 239 | 2.109 | 0.1110 | 2.02 | 2.0695 | 2.06 | 0.1354 | 0.66 | 1139 | 23 | 818 | 5 | 1816 | 37 | 60.86% |
| 16NJ19.15 | 83 | 577 | 0.366 | 0.0937 | 1.56 | 0.9200 | 1.85 | 0.0712 | 1.01 | 662 | 12 | 443 | 4 | 1503 | 24 | 50.59% |
| 16NJ19.16 | 26 | 166 | 0.185 | 0.0734 | 2.18 | 0.9790 | 2.43 | 0.0966 | 0.79 | 693 | 17 | 595 | 5 | 1024 | 22 | 83.46% |
